# Supplementary material for: Identification and functional analysis of non-coding regulatory small RNA FenSr3 in Bacillus amyloliquefaciens LPB-18
Source: PeerJ. 2023 May 15;11:e15236. doi: 10.7717/peerj.15236 (PMC10194069; doi:10.7717/peerj.15236)
Supplement: Supplemental Information 4 [file peerj-11-15236-s004.zip › KO/CK-vs-T1_map/map04624.html]

KEGG PATHWAY: Toll and Imd signaling pathway - Reference pathway


|  |  |
| --- | --- |
| **Toll and Imd signaling pathway - Reference pathway** |  |

[
Pathway menu
| Organism menu
| Pathway entry
| Show description
| User data mapping
]

Insects, such as Drosophila, have evolved complex mechanisms to combat microbial infection. TOLL and IMD pathways are responsible for the immune response that functions through the production of antimicrobial peptides (AMPs). The TOLL pathway mainly detects fungi, Gram-positive bacteria and virulence factors (proteases et al.). Activation of the TOLL pathway results in degradation of Cactus and localization of Dorsal and Dif to the nucleus. On the other hand, the IMD pathway responses to Gram-negative bacteria and leads to the activation of Relish. Dorsal, Dif and Relish induce the transcriptional up-regulation of AMPs in the nucleus.

|  |  |  |
| --- | --- | --- |
| Reference pathway | 184% 150% 122% 100% 82% 67% 55% | 图片下载 |
